# Graphene-Based Dual-Band Metasurface Absorber with High Frequency Ratio

**DOI:** 10.3390/nano14181522

**Published:** 2024-09-20

**Authors:** Anjie Cao, Nengfu Chen, Weiren Zhu, Zhansheng Chen

**Affiliations:** 1 Department of Electronic Engineering, Shanghai Jiao Tong University, Shanghai 200240, China; caj_ddr@sjtu.edu.cn (A.C.);; 2 Shanghai Institute of Satellite Engineering, Shanghai 201109, China; 3 Shanghai Academy of Spaceflight Technology, Shanghai 201109, China

**Keywords:** metasurface, high efficiency, graphene, dual-band

## Abstract

In this paper, we propose a novel dual-band metasurface absorber with a high frequency ratio based on graphene. By carefully designing a centrally symmetrical graphene pattern and positioning it on a glass medium, while utilizing ITO as a ground, the metasurface absorber achieves remarkable high frequency ratio microwave absorption. Specifically, this metasurface absorber exhibits two distinct resonance points at 3.7 GHz and 14 GHz, with an impressive frequency ratio over 3.5. It achieves over 90% absorption efficiency in the frequency ranges of 3.5–4.5 GHz and 13.5–14.5 GHz, highlighting its capability to effectively absorb microwaves across widely spaced frequency bands. Furthermore, the metasurface absorber demonstrates optical transparency and polarization insensitivity, adding to its versatility and potential applications. The measured results of the fabricated prototype validate its design and potential for practical use.

## 1. Introduction

Metasurfaces have emerged as a compelling research area in electromagnetics and optics, offering a unique paradigm for manipulating electromagnetic waves with subwavelength-scale structures [1,2,3,4,5]. Composed of engineered arrays of subwavelength elements, metasurfaces enable precise control over the phase [6], amplitude [7], and polarization [8] of electromagnetic waves. Through careful design, metasurfaces can shape wavefronts [9], steer beams [10], and realize functionalities traditionally achieved with bulk components. Metasurfaces find applications in imaging [11], sensing [12], and communication [13], being promising compact and lightweight devices with unprecedented functionalities. The development of metasurfaces has opened up exciting avenues for advancing microwave technologies and has spurred significant research efforts toward harnessing their potential in various fields [14,15].

Graphene, a single layer of carbon atoms arranged in a two-dimensional hexagonal lattice, has garnered significant attention in the scientific community due to its exceptional properties [16,17,18,19]. Particularly, graphene-based metasurfaces offer exciting prospects for advanced electromagnetic wave manipulation [20,21,22]. Their tunable and dynamic conductivity, high carrier mobility, and strong interaction with light make them an ideal candidate for metasurface applications. Graphene-based metasurfaces provide unprecedented control over light-matter interactions, enabling functionalities such as beam steering, polarization control, and wavefront manipulation across various wavelengths [23,24,25,26]. Additionally, graphene’s broadband absorption enhances electromagnetic wave or light trapping and absorption, making it valuable for applications such as photodetectors [27,28,29] and energy harvesting devices [30,31]. The integration of graphene in metasurfaces opens avenues for compact and versatile devices with enhanced performance.

Designing high-frequency-ratio dual-band absorption metasurfaces is challenging due to the need for precise control over structural parameters and the trade-off between absorption efficiency and bandwidth. Achieving efficient absorption at two widely separated frequencies demands intricate optimization of subwavelength structures. It requires exploring new design principles, advanced optimization algorithms, and innovative fabrication techniques. Overcoming these challenges is crucial for unlocking the full potential of high-frequency-ratio dual-band absorption metasurface absorbers in various applications such as multispectral sensing, energy harvesting, thermal management, communication systems, and photonic devices. High-frequency-ratio dual-band absorption metasurface absorbers offer enhanced sensitivity, expanded bandwidth, and versatile functionality.

In this paper, we propose a graphene-based metasurface absorber featuring a centrally symmetric patterned graphene as the top layer, quartz glass as the middle substrate, and an ITO film as the back plate. The resonance principles of this metasurface absorber are numerically demonstrated through CST Microwave Studio, where the dual-band absorption with a high frequency ratio is clearly achieved. The patterned graphene surfaces of the metasurface absorber are successfully fabricated using methods based on chemical vapor deposition and laser etching. The performance of the proposed metasurface absorber is experimentally verified.

## 2. Design and Simulation

According to the Kubo formula [32], graphene’s the surface conductivity has both intraband and interband contributions, as follows:

(1) $$\sigma_{\mathrm{intra}}\left(\omega ,\mu_{c},\Gamma ,T\right)=\frac{ie^{2}k_{B}T}{\pi \hbar^{2}\left(\omega +2i\Gamma \right)}\left\{\frac{\mu_{c}}{k_{B}T}+2\ln\left[\exp\left(-\frac{\mu_{c}}{k_{B}T}\right)+1\right]\right\},$$

and

(2) $$\sigma_{\mathrm{inter}}\left(\omega ,\mu_{c},\Gamma ,T\right)=\frac{ie^{2}}{4\pi \hbar }\ln\left(\frac{2|\mu_{c}|-\omega -2i\Gamma }{2|\mu_{c}|+\omega +2i\Gamma }\right).$$

Here, $k_{B}$ is the Boltzmann constant and *ℏ* is the Plank constant, Γ=1/(2τ), with τ being the carrier relaxation time in graphene, and $\mu_{c}$ being graphene’s chemical potential.

In the microwave band, graphene’s surface conductivity is mainly defined by intraband transitions [7]. Therefore, at microwave frequencies, graphene can be modeled as a resistive planar sheet of resistance [33],

(3) $$R_{g}=\frac{1}{\sigma_{g}}\approx \frac{1}{\sigma_{\mathrm{intra}}}.$$

The sheet resistance of graphene is related to the chemical potential and the relaxation time of graphene.

**Figure 1:**
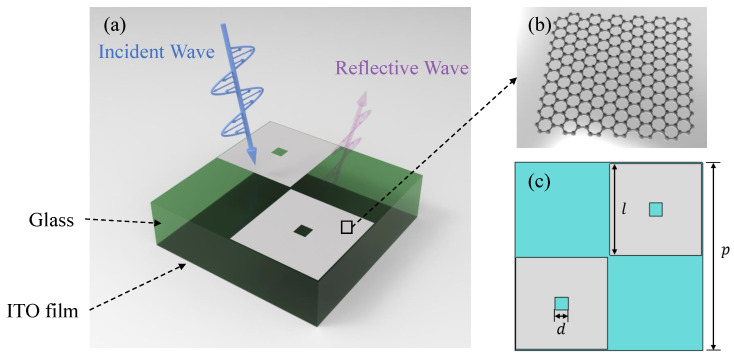
(**a**) Schematic view of the proposed metasurface absorber, (**b**) graphene, and (**c**) geometric parameters of the metasurface absorber.

The unit cell structure of the metasurface absorber is shown in Figure 1a. The overall structure consists of three layers: The top layer consists of a segmented graphene surface with a surface resistance of 90 Ω/sq. The intermediate dielectric layer is composed of glass with a dielectric constant of 7.6, and its thickness is 6 mm. The bottom layer is constructed from an ITO ground, featuring a surface resistance of 6 Ω/sq. Owing to the optically transparent attributes inherent in all three constituent layers, the resultant metasurface structure attains optical transparency. The dimensions of the top graphene layer are configured as illustrated in Figure 1c. The bottom left and top right sections depict identically sized, mutually independent graphene cross-shaped patches, thereby satisfying a condition of central symmetry. Conversely, the top left and bottom right sections remain devoid of any structural elements. The geometric parameters are defined as follows: p=22 mm, l=10.9 mm, d=1.5 mm, and the thickness of the glass layer is 6 mm. It is worth noting that, since the thickness of monolayer graphene is sub-nanometer while the typical dimension of microwave devices is in mm or cm scale, it may include considerable numerical error when model graphene by its electrical permittivity. Similar to those in [8,11], the graphene layer is modeled as a resistance sheet with zero thickness in our simulation.

**Figure 2:**
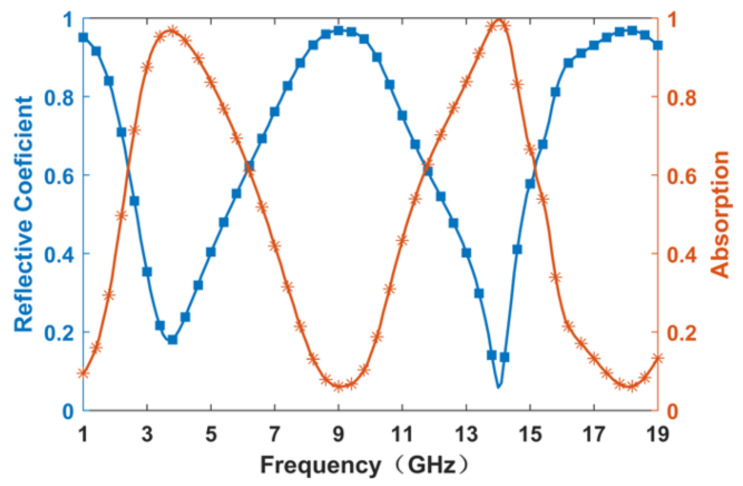
Simulated reflective coefficient and absorptivity curves of the proposed metasurface absorber.

The metasurface absorber is numerically investigated using the commercial software CST Microwave Studio 2019 version. In the simulation, the *x* and *y* directions are set as periodic boundaries, while the *z* direction is set as an open (adding space) boundary condition. Plane waves were employed as the excitation source, and a frequency solver was employed for computations. In our simulation, the distances of the ports from the metasurface were 20 mm for both positive and negative z directions. We set adaptive tetrahedral mesh refinement to reach accurate numerical results. The results are illustrated in Figure 2, depicting the reflection coefficient and absorptivity of the metasurface. The absorptivity at each frequency point was calculated using the following formula, $A\left(\omega \right)=1-{\left|r\left(\omega \right)\right|}^{2}-{\left|t\left(\omega \right)\right|}^{2}$, where |r(ω)| represents the reflection coefficient, |t(ω)| represents the transmission coefficient; moreover, since transmission is negligible within this structure, it can be disregarded. As discerned from the graph, two absorption peaks are evident at 3.7 GHz and 14 GHz, each accompanied by an absorption bandwidth of approximately 1 GHz centered around these two frequency points.

**Figure 3:**
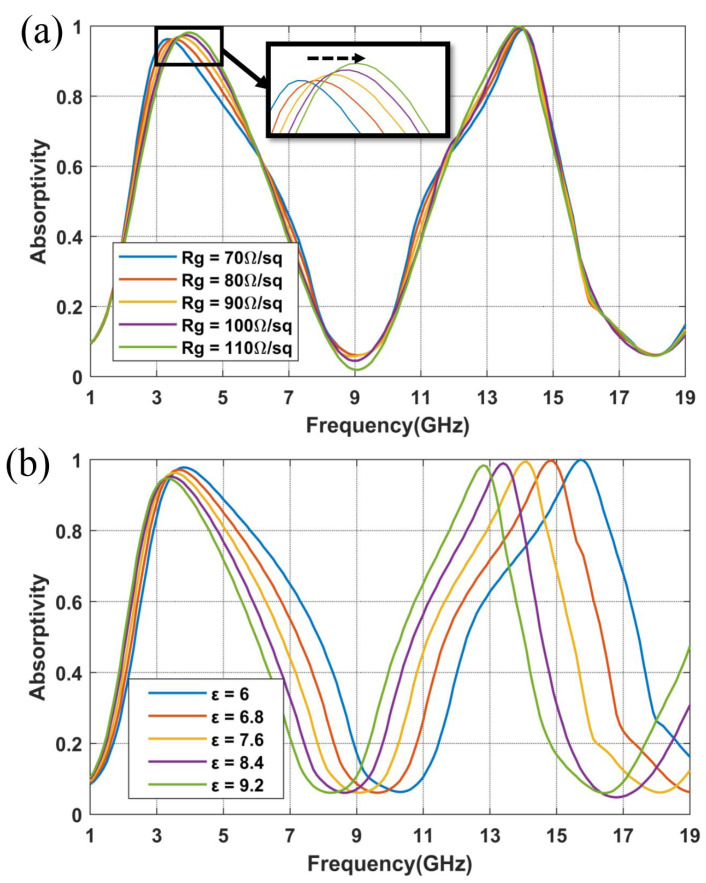
(**a**) Variation of absorptivity curves when adjusting (**a**) surface resistance of graphene $R_{g}$ and (**b**) dielectric constant of the substrate ε.

The material parameters are crucial to the performance of the designed metasurface, such as the surface resistance of graphene $R_{g}$ and the dielectric constant of the substrate ε. This is particularly important considering that, due to limitations imposed by the fabrication process, an exact match between the simulated and actual values of these parameters cannot be guaranteed. Therefore, it is necessary to investigate the impact when varying these crucial material parameters. Figure 3a,b illustrate the impact of variations within a certain range of material parameters, $R_{g}$ and ε, on the absorption efficiency.

As depicted in Figure 3a, the high-frequency point, $f_{2}$, remains relatively stable regardless of the changes in $R_{g}$. Regarding the low-frequency point, $f_{1}$, as the value of $R_{g}$ increases, $f_{1}$ distinctly shifts to the right. Once $R_{g}$ reaches 90 Ω/sq, it no longer satisfies the requirement for 90% absorption at 3.7 GHz. Conversely, when $R_{g}$ decreases, the absorptivity criteria are consistently met. Therefore, the range of graphene surface resistance between 30 and 70 Ω/sq accommodates the design specifications. As indicated by Figure 3b, variations in the dielectric constant of the substrate primarily affect the high-frequency $f_{2}$. It is clearly seen that the resonance frequency $f_{2}$ shifts from 15.8 GHz to 12.7 GHz when the dielectric constant ε increases from 6 to 9.2.

**Figure 4:**
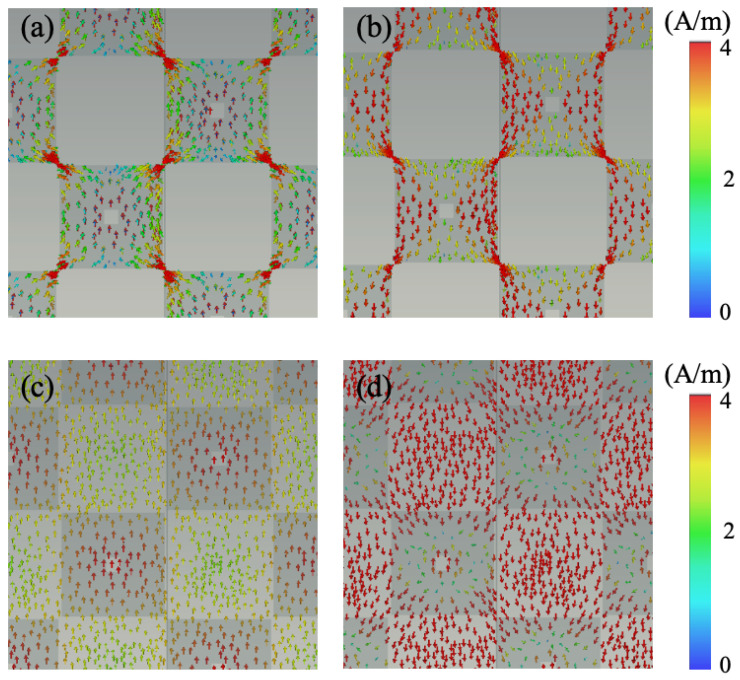
The surface currents of (**a**,**b**) graphene layer and (**c**,**d**) ITO ground at 3.7 GHz and 14 GHz, respectively.

**Figure 5:**
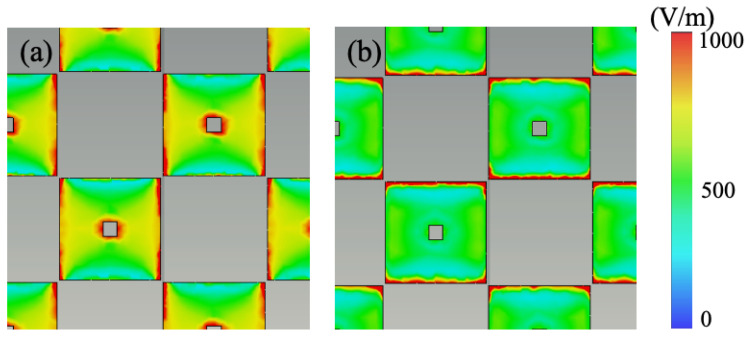
The electric fields in the top view at resonant frequencies of (**a**) 3.7 GHz and (**b**) 14 GHz.

**Figure 6:**
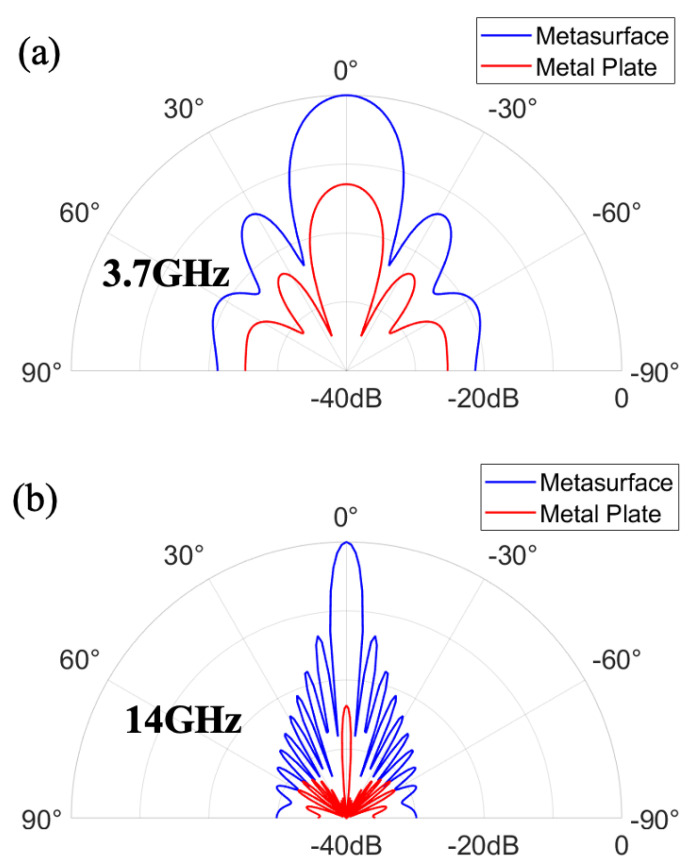
Full-wave simulated scattering patterns at H-Plane at the frequencies of (**a**) 3.7 GHz and (**b**) 14 GHz.

From the perspective of resonance principles, Figure 4a,b present the electric field distribution at two absorption peak frequencies. It is evident from the figure that, for both frequencies, the electric field predominantly concentrates at the four corners of the graphene patches, indicating the occurrence of electric resonance at both peak points. Figure 4a–d depict the surface current distribution of graphene and ITO at the two resonance frequencies. It is observable from the graph that at both resonance points, the direction of surface current on the top graphene layer is opposite to that on the underlying ITO ground, indicating the presence of magnetic resonance. Hence, both absorption peak frequencies in the metasurface absorber originate from the coexistence of electric and magnetic resonances. Moreover, the disparities in electric field distribution and surface current distribution reveal that electric resonance is more pronounced at 3.7 GHz, while magnetic resonance dominates at 14 GHz (Figure 5 and Figure 6).

## 3. Fabrication and Measurement

As the microwave wavelengths are in the centimeter range, large-sized, high-quality monolayer graphene is essential for investigating graphene metasurfaces in the microwave domain. We employ chemical vapor deposition (CVD) on a 25 μm thick copper substrates to synthesize monolayer graphene. This method offers a mature fabrication process that can be continuously improved by refining growth conditions, resulting in the production of continuous, high-quality monolayer graphene films. After CVD preparation, the deposited graphene is then transfered to a 125 μm thick PET film via the dry transfer method. It is worth noting that the monolayer graphene layer was transferred twice, with N-type doping being used to improve its homogeneity and reduce the sheet resistance. Finally, the structured patterns of the graphene layer were fabricated via a standard laser beam cutting method.

**Figure 7:**
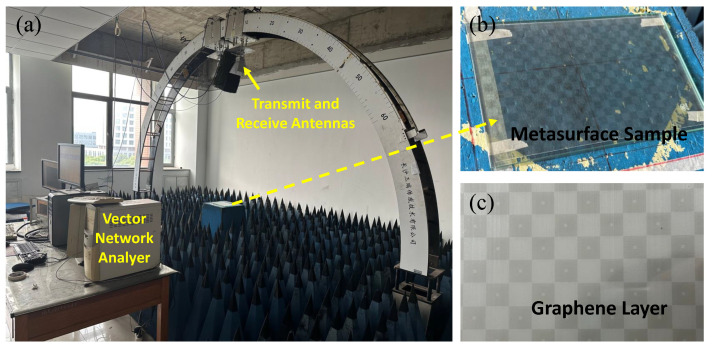
Photograph of (**a**) measurement setup, (**b**) metasurface test sample, and (**c**) graphene patterned layer.

To validate the effectiveness of the designed metasurface absorber, we conducted graphene processing and testing experiments. Based on the material dimensions, we fabricated a 9×13 unit array. The fabricated overall metasurface absorber and the graphene pattern are depicted in Figure 7b,c, respectively. The overall dimension of the metasurface absorber is 20 cm × 30 cm, with a total thickness of around 6.25 mm. The experimental setup is illustrated in Figure 7a, where an arch-shaped structure is employed to secure the far-field reflection with two transmitting and receiving antennas, where the metasurface absorber sample is positioned in between absorptive foams. Before each test, the vector network analyzer is calibrated using a metal plate of the same size as the metasurface absorber sample.

**Figure 8:**
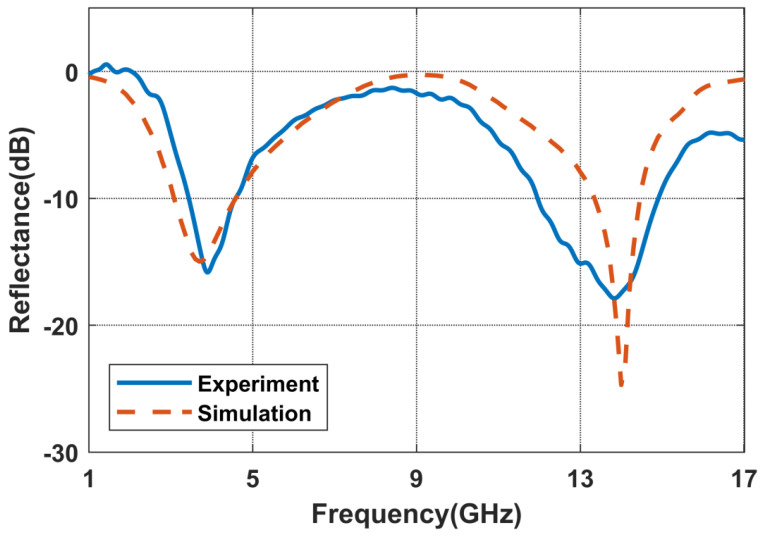
Comparison of the reflection curves of the metasurface absorber via an experiment and simulation.

Under normal incidence, the measured results are presented in Figure 8. There exists a certain disparity between the experimental and simulation results. Specifically, the low-frequency absorption peak is observed at 3.8 GHz, while the high-frequency absorption peak is situated at 14 GHz. Overall, the experimental results are in reasonable agreement with the expected behavior. The deviations in frequency points can primarily be attributed to factors such as imprecise material parameters and insufficient material homogeneity.

## 4. Conclusions

In conclusion, we have numerically designed and experimentally demonstrated a novel dual-band metasurface absorber with a high frequency ratio based on a structured graphene pattern. Such a dual-band metasurface absorber is carefully designed with a centrally symmetrical graphene pattern and placed on a glass medium with an ITO layer as a ground. The dual-band metasurface absorber shows remarkable microwave absorption with a high frequency ratio. Specifically, this metasurface absorber shows two distinct resonance points at 3.7 GHz and 14 GHz, with an impressive frequency ratio over 3.5. It achieves over 90% absorption efficiency in the frequency ranges of 3.5–4.5 GHz and 13.5–14.5 GHz. Furthermore, the metasurface absorber demonstrates optical transparency and polarization insensitivity, adding to its versatility and potential applications. The measured results are in good accordance with the simulation, validating its potential for practical use. 

## Data Availability

The data that support the findings of this study are available from the corresponding author upon reasonable request.
